# Comparison of Findings From Fecal Occult Blood Test and Esophagogastroduodenoscopy With Histopathology Among Symptomatic Saudi Adults: A Retrospective Study

**DOI:** 10.7759/cureus.33119

**Published:** 2022-12-30

**Authors:** Waleed Alhuzaim, Jood Alnojaidi, Shuruq AlKhalaf, Shahad Almalki, Yara Aldosari, Amani Abualnaja

**Affiliations:** 1 Department of Medicine, Imam Mohammad Ibn Saud Islamic University, Riyadh, SAU; 2 Department of Gastroenterology, Imam Mohammad Ibn Saud Islamic University, Riyadh, SAU

**Keywords:** histopathology, egd, esophagogastroduodenoscopy, fobt, fecal occult blood test

## Abstract

Background

A fecal occult blood test is an established way to detect blood in stool samples. However, this diagnostic test is prone to false positives and false negatives, not to mention misuse and misinterpretation of results. In this study, we aimed to compare relevant findings among three diagnostic tests: a fecal occult blood test, an esophagogastroduodenoscopy, and histopathology.

Methods

This study used a retrospective analysis of 74 patients' electronic medical records from September 2021 to September 2022 at the Human Clinic and Gastroenterology Specialized Clinic in Riyadh, Saudi Arabia. Symptomatic adult Saudi patients who underwent a fecal occult blood test, esophagogastroduodenoscopy, and histopathology were included in the study.

Results

A total of 74 patients with a mean age of 43.76 ± 15.1 years were analyzed. More than half of the patients were men (63.5%). Eighteen (18) individuals tested positive through a fecal occult blood test, and 49 individuals showed a positive finding under esophagogastroduodenoscopy. Furthermore, statistical analyses revealed a significant correlation between fecal occult blood test outcomes and histopathological outcomes (p = 0.001).

Conclusion

A significant proportion of the sample population presented a false negative result under the fecal occult blood test. This emphasizes the importance of confirmatory endoscopic procedures and subsequent histopathology in the diagnosis of abnormalities in the upper gastrointestinal tract.

## Introduction

A fecal occult blood test (FOBT) is a diagnostic laboratory test performed to check for the presence of microscopic amounts of blood in the stool [1]. Often used for screening for colorectal polyps or cancer in asymptomatic populations, FOBT proves to be an effective tool if administered correctly; screening with FOBT has shown associations with decreased morbidity and mortality [1]. However, FOBT is non-specific and prone to misinterpretation of findings; this test only detects the presence or absence of blood in the stool, and additional tests are required to determine the source of bleeding [2]. A non-bleeding polyp will return a false-negative result. On the same note, blood from other sources such as hemorrhoids, dietary sources, or even peptic ulcers will return a false positive result, indicating other pathology but not precisely colorectal cancer [2]. Usually, dietary restrictions such as those against red meat and vitamin C-containing diets are put in place before the test [3]. Along with these are medications such as nonsteroidal anti-inflammatory drugs (NSAID) and anticoagulants, which may also return false positive results [3]. A retrospective review of patient charts in Ontario, Canada, concluded that diagnosing symptomatic patients through FOBT was not beneficial; this was attributed to the improper implementation of FOBT in the inpatient setting [3]. They observed a lot of misuse in terms of documentation of diet and medication use, which may lead to longer hospital stays and increased medical costs [3]. A positive result from FOBT may warrant further examination through endoscopy.

Esophagogastroduodenoscopy (EGD) is a diagnostic tool involving endoscopy of the upper gastrointestinal tract. EGD can be used to locate possible upper gastrointestinal sources of bleeding and whether cancer is present in the upper gastrointestinal tract [4]. Additionally, diagnosis and management of some conditions may be performed alongside EGD, for example, by assessing the extent of an injury after caustic ingestion or controlling upper gastrointestinal bleeding [5]. Tissue samples may then be sent to the histopathologist for further examination, diagnosis, and interpretation [6].

In the clinical setting, endoscopic procedures are usually performed after a positive FOBT result. A retrospective analysis of 260 patients who underwent same-day dual endoscopy (colonoscopy and EGD) after a positive FOBT result showed that 52% of the sample population exhibited positive colonoscopy findings, while 16.1% had positive results for EGD [7]. The findings also recommended colonoscopy as the initial endoscopic procedure for the diagnosis of patients who are FOBT positive, and that same-day dual endoscopy is not cost-efficient [7]. Another study published in 2010 aimed to determine the necessity of EGD for patients who are FOBT-positive but colonoscopy-negative [8]. The authors observed 46 EGD-positive individuals among 233 patients; one had angiodysplasia, and 45 patients had peptic ulcers. Their results also revealed that older age, infection with H. pylori, low hemoglobin levels, and alcohol consumption were significant clinical predictors of being EGD-positive [8]. Meanwhile, a systematic review revealed that the currently available evidence is insufficient to conclude whether routine EGD is appropriate for patients who are FOBT-positive but colonoscopy-negative [9]. The authors argue that the clinical judgment on performing EGD is on a case-by-case basis [9]. The majority of the studies available in the literature compare FOBT and endoscopy of the lower gastrointestinal tract. This study aims to contribute to the current literature by comparing relevant findings between FOBT, endoscopy of the upper gastrointestinal tract, and histopathology among symptomatic Saudi adults.

## Materials and methods

Design of the study

This retrospective cohort study was conducted from September 2021 to September 2022 among 74 symptomatic Saudi adult patients from the Human Clinic, a gastroenterology-specialized clinic in Riyadh, Saudi Arabia. Inclusion criteria are as follows: patients aged 18 years and older exhibiting dyspepsia, abdominal pain and/or distention, nausea and vomiting, poor appetite, weight loss, or gastroesophageal reflux who underwent a fecal occult blood test (FOBT), an esophagogastroduodenoscopy (EGD), and histopathological examination of the tissue taken from the EGD. Asymptomatic patients and those less than 18 years old were not included in this study.

Data collection

Patients’ data were collected through their electronic medical records. Relevant demographic and medical information such as age, sex, body mass index (BMI), old or current medications, chronic diseases (e.g., diabetes mellitus, hypertension, and vasculitis), symptoms, FOBT findings, hemoglobin levels, given that the abnormal value is <12 g per dL in non-pregnant women and <13 g per dL in men, with normal values ≥12g per dL and ≥13 g per dL for non-pregnant women and men, respectively, [10], CLO (Campylobacter-like organism) test findings, and EGD and histopathological findings were obtained. Informed consent was obtained from all of the participants. This study was approved by the Institutional Review Board at Imam Mohammad Ibn Saud Islamic University (IRB number 139-2021) in November 2021.

Statistical analysis

Data were analyzed with Statistical Package for the Social Sciences (SPSS) version 23 (IBM Corp., Armonk, N.Y., USA). For categorical and nominal variables, descriptive statistics were used, and data were presented through counts and percentages. Continuous variables were checked for normality, and a comparison of means was performed through parametric or non-parametric tests; these variables were presented through means and standard deviations. This study employed the chi-square test to establish a relationship between categorical variables. To identify significant predictors, a general linear model with interaction was used as a model. Furthermore, a p-value of 0.05 was used to reject the null hypothesis.

## Results

The sample population's demographics and clinical attributes were enumerated in Table 1 and Table 2. Further data on participants’ medications and chronic illnesses are shown in Appendices A and B, respectively.

**Table 1:** Demographic attributes of the population under study (N = 74) Abbreviations: N: number; SD: standard deviation; BMI: body mass index

Variable	N (%) or Mean (SD)	Range
Age	43.76 (15.1)	19 - 94
18-30	14 (18.9)	
31-45	29 (39.2)	
46-60	18 (24.3)	
>60	13 (17.6)	
Gender		
Males	47 (63.5)	
Females	27 (36.5)	
BMI	26.84 (6.2)	13.60 – 45.67
Underweight	6 (8.1)	
Normal	25 (33.8)	
Overweight	19 (25.7)	
Obese	21 (28.4)	
Missing	3 (4.1)	

**Table 2:** Clinical attributes of the population under study Abbreviations: Hgb: hemoglobin; FOBT: fecal occult blood test; EGD: esophagogastroduodenoscopy; CLO: Campylobacter-like organism

		N (%)
Hgb	Normal	18 (24.3)
	Abnormal	24 (32.4)
	Missing	32 (43.2)
CLO test	Negative	43 (58.1)
	Positive	12 (16.2)
	CLO test not performed	6 (8.1)
	Missing	13 (17.6)
Chronic diseases	With chronic diseases	20 (27.0)
	Without chronic diseases	54 (73.0)
FOBT	Negative	56 (75.7)
	Positive	18 (24.3)
EGD findings	Negative	25 (33.8)
	Positive	49 (66.2)
Histopathology	No biopsy/pathology	49 (66.2)
	With histopathology	25 (33.8)

**Figure 1:**
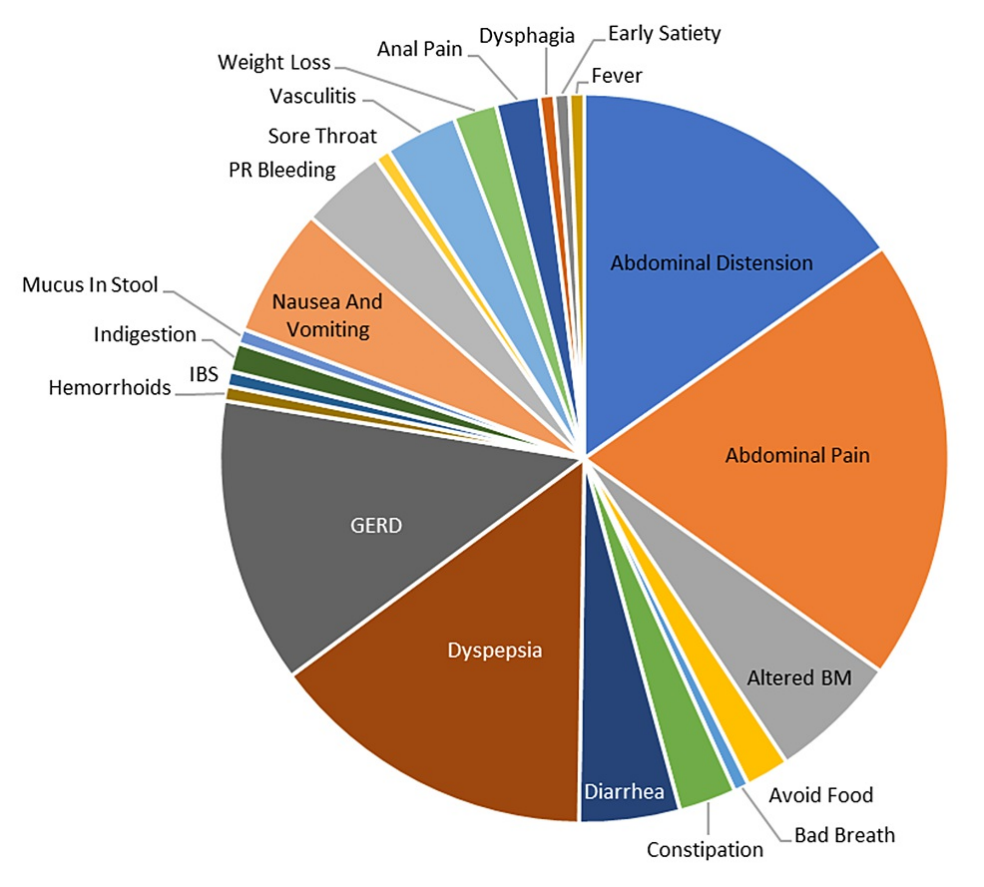
Symptoms experienced by the population under study

The symptoms experienced by the sample population under study are summarized in Figure 1. The top symptoms were abdominal pain, which was seen in 31 (41.9%) participants, while 24 (32.4%) experienced abdominal distension, followed by dyspepsia and gastroesophageal reflux disease (GERD) in 23 (31.1%) and 20 (27.0%) of the participants, respectively.

**Figure 2:**
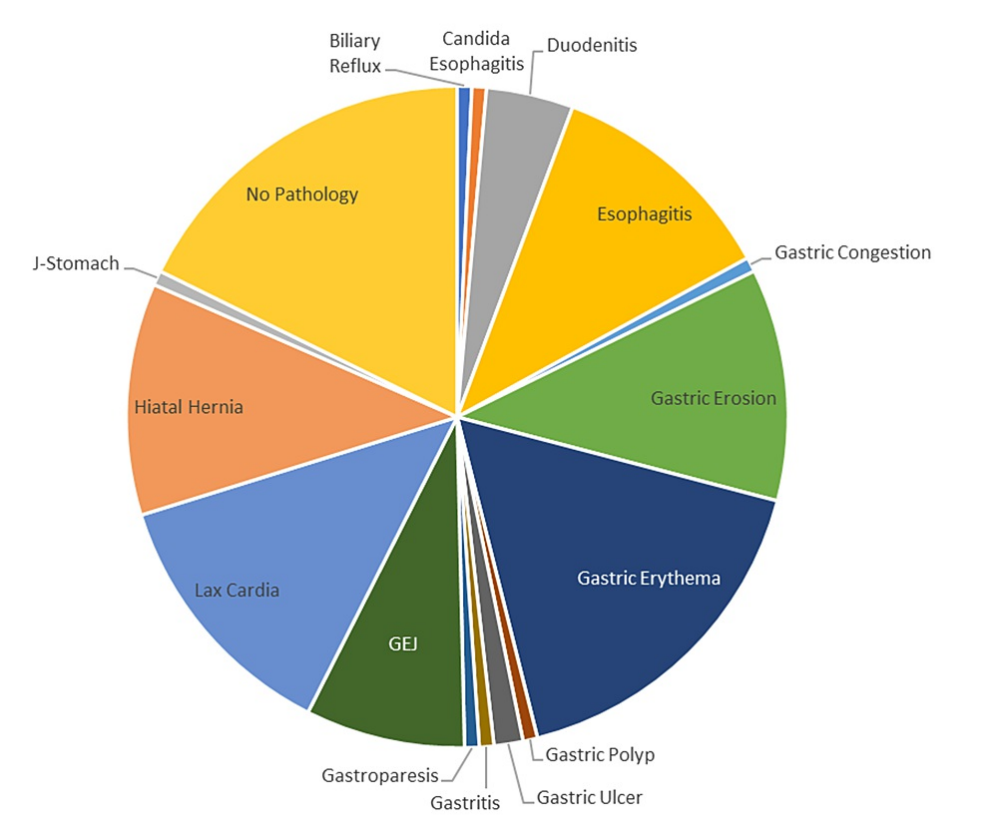
EGD findings on the population under study

All patients underwent EGD; two-thirds (66.2%) of the population exhibited findings, while the rest (33.8%) showed no relevant findings. The most common EGD findings are gastric erythema in 24 participants (32.4%), followed by lax cardia in 18 participants (24.3%), and equal findings of hiatal hernia, gastric erosions, and esophagitis in 16 (21.6%) participants of each. EGD results are summarized in Figure 2.

**Figure 3:**
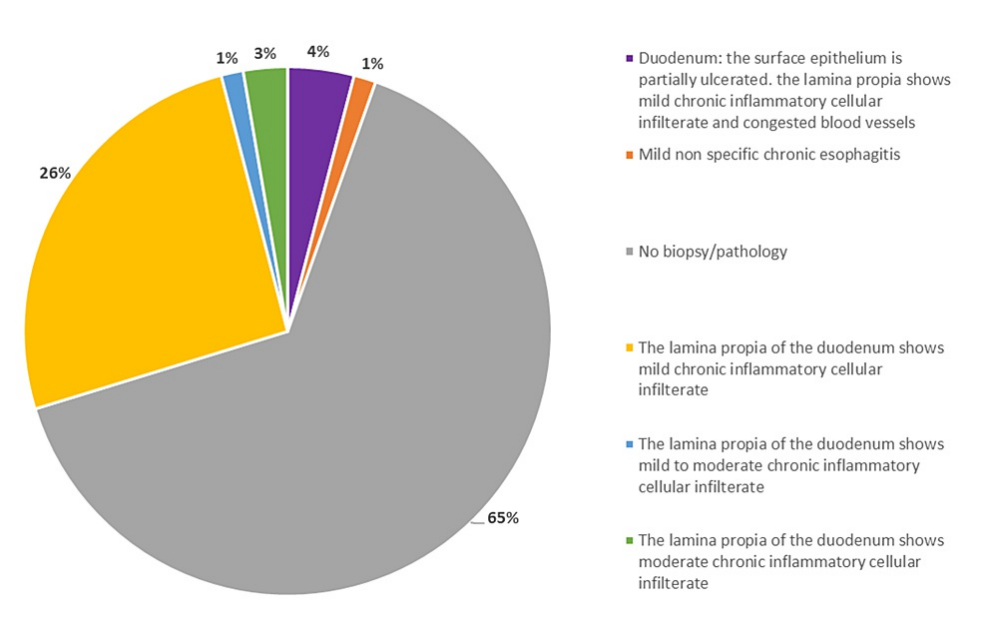
Histopathological findings on the population under study

Positive histopathological results have been determined in 26 (35.1%) patients. Meanwhile, 48 (64.9%) patients exhibited a negative histopathological result. The most common diagnosis suggested a mild chronic inflammatory cellular infiltrate in the lamina propria of the duodenum, occurring in 19 (73.1%) of the patients with abnormal findings. Results from histopathology are summarized in Figure 3.

The comparison of FOBT results with EGD and histopathology results is demonstrated in Table 3. A significant result (p-value 0.001) in the comparison between FOBT and histopathology can be seen. 

**Table 3:** Comparison of FOBT vs. histopathology and EGD results a-significant using the chi-square test at <0.05 level.

Variables		Total	FOBT(+/-)		p-value
			Negative	Positive	
Total		74	56(75.7%)	18(24.3%)	-
Histopathology	No pathology	49	43(87.8%)	6(12.2%)	0.001^a^
	With histopathology	25	13(52.0%)	12(48.0%)	
EGD results, if available	Negative	25	19(76.0%)	6(24.0%)	0.963
	Positive	49	37(75.5%)	12(24.5%)	

A comparison between FOBT vs. histopathology and EGD results filtered by gender in Table 4 showed a significant correlation between FOBT outcomes and histopathological outcomes among males (p = 0.048) and females (p = 0.001) but no significant correlation between FOBT outcomes and EGD results.

**Table 4:** Comparison of FOBT vs. histopathology and EGD results filtered by gender a-significant using the chi-Square test at <0.05 level.

Gender		Total	FOBT(+/-) Male		Total	FOBT(+/-) Female	
			Negative	Positive		Negative	Positive
Total		47	36(76.6%)	11(23.4%)	27	20(74.1%)	7(25.9%)
Histopathology	No pathology	29	25(86.2%)	4(13.8%)	20	18(90.0%)	2(10.0%)
	With histopathology	18	11(61.1%)	7(38.9%)	7	2(28.6%)	5(71.4%)
p-value		0.048^a^			0.001^a^		
EGD results, if available	Negative	16	12(75.0%)	4(25.0%)	9	7(77.8%)	2(22.2%)
	Positive	31	24(77.4%)	7(22.6%)	18	13(72.2%)	5(27.8%)
p-value		0.853			0.756		

One more comparison is shown in Table 5 between FOBT vs. histopathology and EGD filtered by age and showed a significant correlation between FOBT outcomes and histopathological outcomes among adults within the 31-45-year-old age group (p < 0.001).

**Table 5:** Comparison of FOBT vs. histopathology and EGD results filtered by age a-significant using the chi-square test at <0.05 level.

Age		Total	FOBT(+/-) <30		Total	FOBT(+/-) 31-45		Total	FOBT(+/-) 46-60		Total	FOBT(+/-) >60	
			Negative	Positive		Negative	Positive		Negative	Positive		Negative	Positive
Total		14	8(57.1%)	6(42.9%)	29	23(79.3%)	6(20.7%)	18	14(77.8%)	4(22.2%)	13	11(84.6%)	2(15.4%)
Histopathology	No pathology	5	3(60.0%)	2(40.0%)	22	21(95.5%)	1(4.5%)	12	10(83.3%)	2(16.7%)	10	9(90.0%)	1(10.0%)
	With histopathology	9	5(55.6%)	4(44.4%)	7	2(28.6%)	5(71.4%)	6	4(66.7%)	2(33.3%)	3	2(66.7%)	1(33.3%)
p-value		0.872			<0.001^a^			0.423			0.326		
EGD results, if available	Negative	3	1(33.3%)	2(66.7%)	9	8(88.9%)	1(11.1%)	8	5(62.5%)	3(37.5%)	5	5(100.0%)	0(0.0%)
	Positive	11	7(63.6%)	4(36.4%)	20	15(75.0%)	5(25.0%)	10	9(90.0%)	1(10.0%)	8	6(75.0%)	2(25.0%)
p-value		0.347			0.393			0.163			0.224		

The comparison between FOBT vs. histopathology and EGD in Table 6 was filtered by BMI and found a significant correlation between FOBT outcomes and histopathological outcomes among those who are obese (p = 0.02).

**Table 6:** Comparison of FOBT vs. histopathology and EGD results filtered by BMI a-significant using the chi-square test at <0.05 level.

BMI		Total	FOBT(+/-) Underweight		Total	FOBT(+/-) Normal		Total	FOBT(+/-) Overweight		Total	FOBT(+/-) Obese	
			Negative	Positive		Negative	Positive		Negative	Positive		Negative	Positive
Total		6	4(66.7%)	2(33.3%)	25	18(72.0%)	7(28.0%)	19	15(78.9%)	4(21.1%)	21	17(81.0%)	4(19.0%)
Histopathology	No pathology	3	3(100.0%)	0(0.0%)	17	14(82.4%)	3(17.6%)	15	13(86.7%)	2(13.3%)	11	11(100.0%)	0(0.0%)
	With histopathology	3	1(33.3%)	2(66.7%)	8	4(50.0%)	4(50.0%)	4	2(50.0%)	2(50.0%)	10	6(60.0%)	4(40.0%)
p-value		0.083			0.093			0.110			0.020^a^		
EGD results, if available	Negative	1	1(100.0%)	0(0.0%)	12	9(75.0%)	3(25.0%)	6	3(50.0%)	3(50.0%)	5	5(100.0%)	0(0.0%)
	Positive	5	3(60.0%)	2(40.0%)	13	9(69.2%)	4(30.8%)	13	12(92.3%)	1(7.7%)	16	12(75.0%)	4(25.0%)
Total		6	4(66.7%)	2(33.3%)	25	18(72.0%)	7(28.0%)	19	15(78.9%)	4(21.1%)	21	17(81.0%)	4(19.0%)
p-value		0.439			0.748			0.035^a^			0.214		

A comparison of FOBT vs. histopathology and EGD results filtered by Hgb was done in Table 7 and showed a significant correlation between abnormal hemoglobin (Hgb) and positive FOBT with histopathology findings (p = 0.003). Furthermore, there was a significant correlation between normal Hgb, negative FOBT, and positive EGD (p = 0.043).

**Table 7:** Comparison of FOBT vs. Histopathology and EGD results filtered by Hgb a-significant using the chi-square test at <0.05 level.

Hgb		Total	FOBT(+/-) Abnormal		Total	FOBT(+/-) Normal	
			Negative	Positive		Negative	Positive
Total		24	17(70.8%)	7(29.2%)	18	14(77.8%)	4(22.2%)
Histopathology	No pathology	17	15(88.2%)	2(11.8%)	9	7(77.8%)	2(22.2%)
	With histopathology	7	2(28.6%)	5(71.4%)	9	7(77.8%)	2(22.2%)
p-value		0.003^a^			>0.999		
EGD results, if available	Negative	5	4(80.0%)	1(20.0%)	3	1(33.3%)	2(66.7%)
	Positive	19	13(68.4%)	6(31.6%)	15	13(86.7%)	2(13.3%)
p-value		0.612			0.043^a^		

The comparison of FOBT vs. histopathology and EGD results filtered by H. pylori is shown in Table 8. The results showed a significant correlation between positive H. pylori results, positive FOBT, and histopathology findings (p = 0.038). 

**Table 8:** Comparison of FOBT vs. histopathology and EGD results filtered by H. pylori results a-significant using the chi-square test at <0.05 level.

H.pylori results		Total	FOBT(+/-) Negative		Total	FOBT(+/-) Positive	
			Negative	Positive		Negative	Positive
Total		43	32(74.4%)	11(25.6%)	12	8(66.7%)	4(33.3%)
Histopathology	No pathology	27	22(81.5%)	5(18.5%)	5	5(100.0%)	0(0.0%)
	With histopathology	16	10(62.5%)	6(37.5%)	7	3(42.9%)	4(57.1%)
p-value		0.168			0.038^a^		
EGD results, if available	Negative	9	5(55.6%)	4(44.4%)	3	2(66.7%)	1(33.3%)
	Positive	34	27(79.4%)	7(20.6%)	9	6(66.7%)	3(33.3%)
p-value		0.145			>0.999		

In Table 9, a comparison of FOBT vs. histopathology and EGD filtered by top symptoms presented by the sample population shows that most of the population presented with one or two of the top symptoms listed in Figure 1. The results demonstrate a significant correlation between positive FOBT and histopathology findings in patients who presented with two top symptoms (p = 0.007).

**Table 9:** Comparison of FOBT vs. histopathology and EGD results filtered by top symptoms a- top symptoms: abdominal distension, abdominal pain, altered BM, diarrhea, dyspepsia, GERD, nausea, and vomiting. b- significant using the chi-square test at <0.05 level.

Top symptoms^a^		Total	FOBT(+/-) None		Total	FOBT(+/-) 1		Total	FOBT(+/-) 2		Total	FOBT(+/-) 3 or more	
			Negative	Positive		Negative	Positive		Negative	Positive		Negative	Positive
Total		6	5(83.3%)	1(16.7%)	31	23(74.2%)	8(25.8%)	26	19(73.1%)	7(26.9%)	11	9(81.8%)	2(18.2%)
Histopathology	No pathology	4	4(100.0%)	0(0.0%)	23	19(82.6%)	4(17.4%)	15	14(93.3%)	1(6.7%)	7	6(85.7%)	1(14.3%)
	With histopathology	2	1(50.0%)	1(50.0%)	8	4(50.0%)	4(50.0%)	11	5(45.5%)	6(54.5%)	4	3(75.0%)	1(25.0%)
p-value		0.121			0.069			0.007^b^			0.658		
EGD results, if available	Negative	1	1(100.0%)	0(0.0%)	11	7(63.6%)	4(36.4%)	9	8(88.9%)	1(11.1%)	4	3(75.0%)	1(25.0%)
	Positive	5	4(80.0%)	1(20.0%)	20	16(80.0%)	4(20.0%)	17	11(64.7%)	6(35.3%)	7	6(85.7%)	1(14.3%)
p-value		0.642			0.319			0.186			0.658		

A comparison of FOBT vs. histopathology and EGD filtered by chronic disease is shown in Table 10. Fifty-four out of a total of 74 people in the sample population have no chronic diseases; thus, there is a significant correlation between a negative FOBT and, accordingly, no histopathology findings (p = 0.005).

**Table 10:** Comparison of FOBT vs. histopathology and EGD results filtered by chronic diseases a-significant using the chi-square test at <0.05 level.

Chronic diseases		Total	FOBT(+/-) No chronic disease		Total	FOBT(+/-) With chronic diseases	
			Negative	Positive		Negative	Positive
Total		54	39(72.2%)	15(27.8%)	20	17(85.0%)	3(15.0%)
Histopathology	No pathology	34	29(85.3%)	5(14.7%)	15	14(93.3%)	1(6.7%)
	With histopathology	20	10(50.0%)	10(50.0%)	5	3(60.0%)	2(40.0%)
p-value		0.005^a^			0.071		
EGD results, if available	Negative	20	14(70.0%)	6(30.0%)	5	5(100.0%)	0(0.0%)
	Positive	34	25(73.5%)	9(26.5%)	15	12(80.0%)	3(20.0%)
p-value		0.780			0.278		

## Discussion

Given that fecal occult blood test as a diagnostic tool is prone to misuse and misinterpretation of results, this study aimed to compare findings between fecal occult blood test (FOBT), esophagogastroduodenoscopy (EGD), and histopathology among symptomatic Saudi adults. Relevant demographic and medical data were retrieved from electronic patient records.

More than half of the sample population was male (63.5%). The mean age for the study was 43.76 ± 15.1 years, with most of the patients (39.2%) falling under the 31-45 age group. Most of the patients (58.1%) tested negative for H. pylori infection. In terms of hemoglobin levels, 24 patients had abnormal levels while 18 exhibited normal levels. In a published study that aimed to profile the implementation of FOBT in hospitals in Ontario, the mean age of their sample population was 49 years, with the majority being female patients (52%) [3]. Another study on 397 patients who tested positive under immunochemical FOBT revealed a mean age of 50.5 years, with the majority being male (61.5%) [8].

The most common symptoms experienced by the population under study were abdominal pain (41.9%), abdominal distention (32.4%), dyspepsia (31.1%), and gastroesophageal reflux disease (GERD) (27.0%). In the Canadian study, the most common symptom observed was anemia of an undetermined type, followed by hematochezia [3]. Statistical analyses from this study suggested that FOBT outcomes were significantly correlated (p = 0.008) with histopathological outcomes for patients with any two of the most common symptoms (abdominal distention, abdominal pain, altered bowel movements, dyspepsia, GERD, and nausea and vomiting).

In our study, no significant correlation (p=0.963) was established between FOBT outcomes and EGD outcomes. However, a significant correlation between FOBT outcomes and EGD outcomes was documented among patients who were overweight (p=0.035) and among those with normal hemoglobin levels (p=0.043). Studies have emphasized the importance of endoscopy of the upper gastrointestinal tract for patients who are FOBT-positive and colonoscopy-negative [8-11]. For example, in the study by Chiang et al. (2010), 46 out of 233 patients who were FOBT-positive and colonoscopy-negative were EGD-positive, mostly for peptic ulcer diseases [8]. Another study showed that 44 out of 95 patients who were FOBT-positive and colonoscopy-negative were EGD-positive [12]. The study by Chiang et al. concluded that older age, low hemoglobin levels, H. pylori infection, and alcohol intake were significantly associated with a positive EGD finding [8]. In this study, a significant proportion of the patients were FOBT-negative and EGD-positive, suggesting the prevalence of false-negative cases.

On the other hand, the results of our current study establish a significant correlation (p=0.001) between FOBT outcomes and histopathological outcomes. Specifically, a significant correlation between FOBT outcomes and histopathological outcomes was observed among males (p=0.048) and females (p=0.001), among adults within the 31-45-year-old age group (p < 0.001), among those who are obese (p=0.02), among those with abnormal hemoglobin levels (p=0.003), among those who tested positive for H. pylori (p=0.038), among those who suffer from exactly two top symptoms (p=0.007), and among those without chronic diseases (p=0.005), were observed. Histopathology is regarded as the gold standard in terms of diagnosing pathological lesions in the lower digestive tract [13]. In this study, a proportion of patients were FOBT-negative with a particular histopathological finding, which further confirmed the prevalence of false-negative cases in the sample population.

False-negative results under FOBT can be caused by several reasons. A study published in 2013 noted that patients who were smokers and those who were older had a significantly higher chance of generating a false negative result under immunochemical FOBT [14]. Another study found that males, those with hyperglycemia, hypertension, and obesity, as well as those who smoked and had a family history of colorectal cancer, were more likely to have false-negative results in the immunochemical FOBT [15]. Ingestion of ascorbic acid has also been observed to contribute to a false-negative result [16]. This is due to the inhibition of the pseudoperoxidase activity of heme, which occurs in the presence of low levels of ascorbic acid (vitamin C) [16].

Limitations

There are a few limitations to this study. First, there were no previous similar studies to compare the results with those of this study. Second, the participants in this study are from regions of Saudi Arabia, but they do not represent the entire population of Saudi Arabia. Therefore, the study outcome should be extrapolated with caution. Another limitation is that not all the participants did EGD and a biopsy simultaneously, which made it an insufficient sample size for statistical measurements.

## Conclusions

In this study, we compared the relevant findings between fecal occult blood testing (FOBT), esophagogastroduodenoscopy (EGD), and the histopathology of the symptomatic adult Saudi population. Results have shown a significant number of false negative cases under FOBT, as evidenced by the low positivity rate in FOBT and the relatively higher positivity rates in EGD and histopathology. False-negative results are not uncommon in FOBT, and this can be associated with the population's demographics and clinical attributes.

Further studies regarding the association of diagnostic outcomes with population demographics and clinical attributes are recommended. The results of this study also emphasize the significance of a combination of diagnostic tests to fully elucidate the pathology of diseases in the upper gastrointestinal tract.

## Appendices

Appendix A

**Table 11:** Old medications

Medication			
Variables		Count	%
Old medication	Amlodipine	2	2.7
	Amlor	1	1.4
	Arbiten plus	1	1.4
	Aspirin	2	2.7
	Atorvastatin	1	1.4
	Calcium-Sandoz	1	1.4
	Cefix	1	1.4
	Ciprofloxacin	1	1.4
	Clarithromicin	1	1.4
	Copper	1	1.4
	Diamicron	1	1.4
	Diclofenac	1	1.4
	Disprin	1	1.4
	DM meds	1	1.4
	Domperidone	1	1.4
	Dompy	1	1.4
	Dysbiocide	1	1.4
	Esomeprazole	1	1.4
	Exforge	1	1.4
	FDgard	1	1.4
	Fevadol	1	1.4
	Flagyl	1	1.4
	Folic acid	1	1.4
	Glizide	1	1.4
	Gluco tabs	1	1.4
	Glucophage	4	5.4
	Hymox	1	1.4
	Hyoscine	1	1.4
	Ibgard	1	1.4
	Insulin	2	2.7
	Janumet	1	1.4
	Jardiance	1	1.4
	Juspirin	1	1.4
	Lasiz	1	1.4
	Lipitor	2	2.7
	L-thyroxin	2	2.7
	Metoclopramide	1	1.4
	Montelukast	1	1.4
	Mycophenolate	1	1.4
	Nebilet	1	1.4
	Nexium	6	8.1
	Nitrofurantoin	1	1.4
	Olfen	1	1.4
	ON meds	1	1.4
	ORS	1	1.4
	Panadol	1	1.4
	Pantoprazole	2	2.7
	PPI	3	4.1
	Prednisolone	1	1.4
	Psych meds	1	1.4
	Relvar	2	2.7
	Sandimmum-neoral	1	1.4
	Seretide	1	1.4
	Singulair	1	1.4
	Sucralfate	1	1.4
	Symbicort	1	1.4
	Tothema	1	1.4
	Ventolin	1	1.4
	Vitamin	1	1.4
	Vitamin B complex	1	1.4
	Vitamin D	2	2.7
	Voltaren tab	2	2.7
	Xeractan	1	1.4
	Zantac	2	2.7
	Zestril	1	1.4
	Zocor	1	1.4
	N/A	42	56.8

Appendix B

**Table 12:** Chronic diseases

Chronic diseases			
Variables		Count	%
Chronic diseases	Appendix removed	1	1.4
	Asthma	4	5.4
	Celiac	1	1.4
	Diabetes	10	13.5
	Disk	1	1.4
	Fatty liver	1	1.4
	GB resected	1	1.4
	HTN	6	8.1
	Peri-anal sinus	1	1.4
	Thyroid disease	1	1.4
	Uterus resected	1	1.4
	Vasculitis	5	6.8
	NA	54	73.0
